# Anti-NMDA Receptor Encephalitis Presenting as an Acute Psychotic Episode in a Young Woman: An Underdiagnosed yet Treatable Disorder

**DOI:** 10.1155/2014/868325

**Published:** 2014-10-07

**Authors:** Shikma Keller, Pablo Roitman, Tamir Ben-Hur, Omer Bonne, Amit Lotan

**Affiliations:** ^1^Brain Division, Department of Psychiatry, Hadassah-Hebrew University Medical Center, P.O. Box 12000, 91120 Jerusalem, Israel; ^2^Brain Division, Department of Neurology, Hadassah-Hebrew University Medical Center, 91120 Jerusalem, Israel

## Abstract

Anti-NMDA receptor (NMDAR) encephalitis is a recently identified autoimmune disorder with prominent psychiatric symptoms. Patients usually present with acute behavioral change, psychosis, catatonic symptoms, memory deficits, seizures, dyskinesias, and autonomic instability. In female patients an ovarian teratoma is often identified. We describe a 32-year-old woman who presented with acute psychosis. Shortly after admission, she developed generalized seizures and deteriorated into a catatonic state. Although ancillary tests including MRI, electroencephalogram, and cerebrospinal fluid (CSF) analysis were unremarkable, the presentation of acute psychosis in combination with recurrent seizures and a relentless course suggested autoimmune encephalitis. The patient underwent pelvic ultrasound which disclosed a dermoid cyst and which led to an urgent cystectomy. Plasmapheresis was then initiated, yielding partial response over the next two weeks. Following the detection of high titers of anti-NMDAR antibodies in the CSF, the patient ultimately received second line immunosuppressive treatment with rituximab. Over several months of cognitive rehabilitation a profound improvement was eventually noted, although minor anterograde memory deficits remained. In this report we call for attention to the inclusion of anti-NMDAR encephalitis in the differential diagnosis of acute psychosis. Prompt diagnosis is critical as early immunotherapy and tumor removal could dramatically affect outcomes.

## 1. Introduction

In 2005, a syndrome with prominent psychiatric symptoms, memory loss, decrease in level of consciousness, and central hypoventilation was described in young women with ovarian teratoma and antibodies against an antigen highly expressed in the hippocampus. Soon thereafter the target antigen was identified as the* N*-methyl-D-aspartate receptor (NMDAR) of glutamate [1]. The disorder, named “anti-NMDAR encephalitis,” has since been recognized in patients of all ages, but more frequently in young adults and children with or without teratoma [2]. Patients usually present with acute behavioral change, psychosis, and catatonia that evolve to include seizures, memory deficit, dyskinesias, speech problems, and autonomic and breathing dysregulation. When severe, the disorder may become life-threatening and intensive care treatment is warranted [3]. Roughly 5% of patients with this diagnosis eventually die, most often as a result of neurological and autonomic dysfunction.

The case report outlined below discusses the clinical presentation, workup, and treatment of a patient diagnosed with anti-NMDAR encephalitis. Key features of this disorder, such as a clinical presentation dominated by psychosis, are emphasized. This report thus highlights the increasing need for psychiatrists and other relevant medical disciplines to become aware of this underdiagnosed disorder and consider it in their differential diagnosis.

## 2. Case Presentation

A previously healthy 32-year-old woman was referred to our emergency room (ER) following a 5-day history of sleeplessness, agitation, and auditory hallucinations in the form of “telephone ringing.” The family reported that she fainted during a holiday a few days earlier and was referred to the ER at a regional hospital. Following unremarkable neurological examination and head computerized tomography, the patient was discharged. Since the patient's agitation and hallucinations worsened, she underwent psychiatric consultation, in which a psychotic state had been diagnosed and olanzapine prescribed at 5 mg/day. Three days later, due to further exacerbation in her mental state, the patient was referred to our medical center. On examination, the patient was extremely anxious with a feeling of imminent doom. Although she reported constant sounds of bells ringing and of her children playing, she was reasonably oriented to time and place. Neurological examination did not disclose any focal signs and routine laboratory workup was normal. Owing to the growing agitation and psychosis, the patient was admitted to inpatient care.

Upon admission, the patient experienced a generalized tonic-clonic seizure. Subsequent electroencephalogram (EEG) revealed intermittent focal frontal bursts, and phenytoin loading was initiated. Cerebrospinal fluid (CSF) analysis revealed five lymphocytes, no red blood cells, and normal protein and glucose concentrations. Intravenous acyclovir was initiated until obtaining negative PCR results in the CSF. Brain magnetic resonance imaging (MRI) was normal. After two days extensive laboratory examination of serum and CSF for relevant infectious and immune causes was unremarkable. Nevertheless, during the following days the patient's confusion and agitation escalated; she spoke in incomprehensible words, with intermittent bursts of shouting and disorganized behavior. Being unable to follow basic commands and being extremely negativistic, the patient refused to eat or drink and a nasogastric tube was inserted. Intermittent facial twitching was observed. Due to frequent fluctuations in mental state, seclusion and restraining were required to ensure the patient's safety, despite treatment with olanzapine at 20 mg/day. In an attempt to treat what appeared as a state of extreme catatonic excitation, an escalating regimen of intravenous diazepam was added. Despite phenytoin and diazepam treatment, the patient experienced a second generalized tonic-clonic seizure, and sodium valproate was added to prevent further seizures.

Given the acute presentation of a severe psychotic state, with cognitive impairment, fluctuating behavioral changes, and recurrent seizures despite antiepileptic treatment, a clinical diagnosis of probable autoimmune encephalitis was made, and anti-NMDAR encephalitis was suspected at the top of the differential diagnosis. Hence, CSF and serum samples were shipped to the August Pi i Sunyer Biomedical Research Institute in Barcelona, Spain, for serological testing of anti-NMDAR antibodies. As the current management guidelines for this disorder advocate early detection and removal of a teratoma [3], a pelvic ultrasound was performed. Following detection of a 5 cm dermoid cyst, the patient underwent an urgent laparoscopic cystectomy. As prompt initiation of immunotherapy has been recently shown to predict better outcome [3], the patient was started on plasmapheresis twice a week for a total of five treatments. Ten days later, CSF serologic results returned, demonstrating strong reactivity with the NMDAR in two different techniques, thus confirming the diagnosis of anti-NMDAR encephalitis.

Following plasmapheresis, the patient's mental status improved slightly over the next three weeks, fluctuating between communicable periods, catatonia, and extreme agitation. As the patient's response to the first-line therapy was deemed to be partial and given the recent data suggesting that second line immunosuppressive treatment offered additional benefit in this scenario, the patient was treated with two 1 g rituximab injections two weeks apart. Over the following month, gradual improvement was observed. As the patient's behavior became less disorganized and agitated, antipsychotic and anxiolytic drugs were tapered down. Three months after the onset of symptoms, the patient regained her capacity to perform basic activities of daily life, although executive functions remained relatively poor. Therefore, after discharge, the patient was referred to a cognitive rehabilitation day service.

Over the next three months, the patient regained skills such as planning ahead, taking care of her children and home, and monetary management. On a recent follow-up visit, the patient reported feeling happy and satisfied with her recovery. Together with resuming some of her prior family roles, she considered returning part time to her previous job as a financial bookkeeper. Upon examination, her affect was full and appropriate, with a logical and coherent thought process. Although episodic memory pertaining to events prior to her hospitalization was preserved, she was entirely amnestic for all events that occurred during the three months thereafter. Moreover, a mild, albeit highly troublesome, anterograde memory deficit was still apparent.

## 3. Discussion

This report illustrates the case of a woman admitted following an abrupt-onset episode of hallucinations and confusion. Eventually, the appearance of fluctuations in mental status combined with repeated generalized seizures led the medical staff to suspect the diagnosis of anti-NMDAR encephalitis. The discovery of a teratoma by simple pelvic sonography soon after provided a very strong clue to the diagnosis, leading to urgent removal of the teratoma, even before results of anti-NMDAR antibodies in CSF were obtained. Notably, early removal of the teratoma served as a first-line therapy, with rapid institution of immunosuppressive treatment following the surgery. These interventions resulted in marked improvement over several months' time.

Anti-NMDA-R encephalitis is an autoimmune disorder with a complex presentation, including psychiatric symptoms, memory deficits, and autonomic instability [2]. Roughly 80% of patients are females, with the majority presenting during early adulthood. In nearly half of female patients an underlying neoplasm, most commonly ovarian teratoma, is identified. Based on its immune pathogenesis, early removal of an existing teratoma followed by immunotherapy is the mainstay of treatment. Recent data suggest that over half of patients respond to first-line immunotherapy (steroids, intravenous immunoglobulin, and plasmapheresis, alone or in combination) within four weeks. Moreover, second-line treatment (rituximab and/or cyclophosphamide) is usually effective when first-line therapies fail. When relevant, rapid removal of an associated neoplasm is warranted, possibly reducing future risk for relapse, which may exceed 10% during the first two years. Despite optimal treatment, the recovery process can continue for over 18 months [3].

While the majority of patients present with a combination of behavioral, cognitive, and motor symptoms, as well as speech disorder, seizures, and decreased level of consciousness, psychiatric symptoms often predominate in the early phases. Such a presentation often leads patients to first seek psychiatric evaluation and treatment, causing a crucial delay in diagnosis and institution of immunotherapy [4]. Moreover, a recent large cohort-based study revealed that 4% of patients presented with isolated psychiatric episodes (pure psychiatric symptoms without neurological involvement) [5]. In this cohort MRI of the brain, EEG and CSF studies were abnormal in 33%, 90%, and 79% of patients, respectively. Moreover, results of these ancillary tests in patients with isolated psychiatric episodes were similar to the cohort population at large. As early recognition of these episodes and institution of appropriate therapy was shown to be an important prognostic factor, the authors recommended that, in patients with new onset psychosis, history of encephalitis, subtle neurological symptoms and abnormal, albeit non-specific, CSF, EEG, or MRI findings, prompt screening for NMDAR antibodies, and ovarian teratoma when applicable, should be performed [5].

In summary, the present case illustrates the pertinent need for psychiatrists, neurologists, and other emergency-room physicians to become aware of anti-NMDA-R encephalitis. Although the typical presentation involves a combination of behavioral, cognitive, and motor symptoms, isolated psychiatric episodes can occur. The inclusion of this disorder in the differential diagnosis is critical, as prompt initiation of immunotherapy and tumor removal, if appropriate, could dramatically affect outcome.
